# Influenza-associated acute respiratory deterioration fulfilling acute exacerbation criteria in hospitalized patients with interstitial lung disease: a single-center retrospective cohort study

**DOI:** 10.1186/s12890-026-04561-z

**Published:** 2026-08-07

**Authors:** Hiroshi Nakahama, Kazumasa Ogawa, Atsushi Miyamoto, Atsuko Kurosaki, Yui Takahashi, Takahiro Mitsumura, Shigeo Hanada, Meiyo Tamaoka

**Affiliations:** 1 Department of Respiratory Medicine, Respiratory Center, Toranomon Hospital, 2-2-2 Toranomon, Minato-ku, Tokyo, 105-8470 Japan; 2 Department of Internal Medicine, Nomura Hospital, 8-3-6 Shimo-Renjaku, Mitaka City, Tokyo, 181-8503 Japan; 3 Department of Diagnostic Radiology, Fukujuji Hospital, Japan Anti- Tuberculosis Association, 3-1-24 Matsuyama, Kiyose-shi, Tokyo, 204-8522 Japan

**Keywords:** Acute exacerbation, AE, Interstitial lung disease, ILD, Influenza virus

## Abstract

**Background:**

Acute exacerbation (AE) of interstitial lung disease (ILD) is life threatening, but data on acute respiratory deterioration fulfilling AE criteria after laboratory-confirmed influenza infection are limited. The current study investigated the incidence, clinical features, and outcomes of such events after laboratory-confirmed influenza in hospitalized patients with ILD.

**Methods:**

We retrospectively reviewed adults with pre-existing ILD who were hospitalized with influenza at Toranomon Hospital between April 2014 and April 2019. Influenza was confirmed by rapid diagnostic tests. Fulfillment of the AE criteria was confirmed by two pulmonologists and one radiologist. Baseline characteristics and outcomes were compared between patients with and without AE-criteria events. The Mann–Whitney U test or Fisher’s exact test were used for between-group comparisons, and the Kaplan–Meier method and the log-rank test were used for survival analysis.

**Results:**

Of 927 patients hospitalized with ILD during the study period, 47 had laboratory-confirmed influenza, and 10 of these patients (21.3%) developed AE-criteria events. The median interval from influenza diagnosis to event onset was 5.0 days. Compared with patients without AE-criteria events, those with AE-criteria events had lower percent predicted forced vital capacity (70.0% versus 88.0%, *p* = 0.041) and higher ILD-GAP stage. The in-hospital mortality rate in patients with AE-criteria events was 30.0%. Overall survival from influenza diagnosis appeared shorter in patients with AE-criteria events in unadjusted analysis.

**Conclusions:**

Acute respiratory deterioration fulfilling AE criteria occurred in approximately one fifth of hospitalized patients with ILD after laboratory-confirmed influenza and was associated with high in-hospital mortality. Unadjusted analysis also suggested shorter overall survival in these patients. These findings should be considered exploratory and hypothesis-generating, and require confirmation in larger multicenter studies.

## Background

Interstitial lung disease (ILD) encompasses a heterogeneous group of diffuse parenchymal lung diseases, all characterized by varying degrees of inflammation and fibrosis within the lung interstitium. It can be classified as idiopathic interstitial pneumonia (IIP) or secondary forms associated with specific conditions such as connective tissue disease (CTD), hypersensitivity pneumonitis (HP), drug-induced lung injury, and occupational exposure.

Acute exacerbation (AE) of idiopathic pulmonary fibrosis (IPF) is defined by the revised criteria proposed by the International Working Group report [1] as follows: “Cases which show sudden worsening of respiratory symptoms within one month, with new bilateral ground-glass opacities, or consolidation on high-resolution computed tomography (HRCT), in which cardiac failure or fluid overload are excluded as the primary cause”. AE of IPF has a poor prognosis, with in-hospital mortality rates of approximately 30%–50%.

Patients with chronic lung diseases, including ILD, are particularly vulnerable to severe complications owing to their reduced pulmonary reserve. Viral infections have been implicated as potential triggers of AE [2]. AE triggered by seasonal influenza infection is occasionally encountered in daily clinical practice. However, evidence regarding this phenomenon is limited, with research primarily confined to isolated case reports describing such associations [3]. Current clinical practice guidelines recommend performing annual influenza vaccination in patients with IPF to reduce the risk of AE; however, data directly linking vaccination to reduced AE risk remains limited [4, 5].

The aim of the current study was to investigate the incidence, clinical features, and outcomes of acute respiratory deterioration fulfilling AE criteria in hospitalized patients with ILD following laboratory-confirmed influenza infection. We also compared baseline clinical characteristics between patients with and without AE-criteria events.

## Methods

### Study participants

The study enrolled eligible patients admitted to Toranomon Hospital between April 2014 and April 2019. The inclusion criteria were adults (≥ 18 years) with a pre-existing diagnosis of ILD and radiologic and/or histopathologic evidence of chronic fibrosis. ILD was defined based on clinical, radiological, and/or histopathological findings, as documented in medical records. Diagnoses included IPF, IIP other than IPF, CTD-ILD, drug-induced ILD, and fibrotic HP. Influenza infection was diagnosed using the ESPLINE Influenza A&B-N rapid influenza diagnostic test (Fujirebio Inc., Tokyo, Japan) performed on nasopharyngeal swab specimens.

### Study design

This single-center, retrospective cohort study was conducted in accordance with the Declaration of Helsinki and was approved by the institutional ethics committee of Toranomon Hospital (approval number 1961). Patients were classified into the AE-criteria event group or non-event group based on whether they developed acute respiratory deterioration fulfilling AE criteria within 1 month of influenza diagnosis. AE criteria were based on those proposed by Collard et al. [1], requiring acute worsening of dyspnea within 1 month, new bilateral ground-glass opacities and/or consolidation on HRCT, and exclusion of heart failure or fluid overload as the primary cause. Under the revised AE framework, infection was not considered an exclusion criterion. Therefore, events were classified based on fulfillment of the AE criteria rather than being attributed definitively to influenza-triggered AE-ILD. In the patients with AE-criteria events, fulfillment of the AE criteria was confirmed by two pulmonologists and one radiologist.

### Data collection and outcomes

Variables recorded at the time of influenza diagnosis and hospitalization included age, sex, body mass index (BMI), smoking history, comorbidities, vital signs, arterial blood gas values, and serum biomarkers (including Krebs von den Lungen-6 [KL-6], surfactant protein D [SP-D], and surfactant protein A [SP-A]). Pulmonary function test results (vital capacity [VC], percent predicted VC [%VC], forced vital capacity [FVC], percent predicted FVC [%FVC], carbon monoxide diffusion capacity [DLco], and percent predicted DLco [%DLco]), and the 6-minute walk test results were obtained from the most recent assessments performed within 1 year before influenza diagnosis.

Treatment-related variables included antifibrotic or corticosteroid use, seasonal influenza vaccination status (including timing within the same season), influenza virus type, and antiviral therapy (oseltamivir, peramivir, baloxavir, or laninamivir). Information on polymerase chain reaction (PCR) testing for influenza and other respiratory viruses was extracted from the medical records. We also reviewed available microbiological data, including sputum and blood cultures, urinary antigen tests, bronchoalveolar lavage (BAL) findings, and other investigations for respiratory pathogens when performed as part of routine clinical care.

HRCT patterns were classified based on the 2022 American Thoracic Society/European Respiratory Society/Japanese Respiratory Society/Asociación Latinoamericana de Tórax clinical practice guidelines for IPF [6]. HRCT patterns were evaluated by two pulmonologists and one radiologist, with any discrepancies resolved by consensus. In patients with AE-criteria events, HRCT findings at the time of the events were reviewed, including new ground-glass opacity, consolidation, or mixed ground-glass opacity and consolidation. The distribution of new opacities was classified as peripheral, multifocal, or diffuse according to the HRCT pattern classification proposed by Akira et al. The peripheral pattern was defined as new opacities predominantly adjacent to pre-existing subpleural fibrotic lesions, the multifocal pattern as multiple but limited areas of new opacities involving both central and peripheral lung regions, and the diffuse pattern as widespread bilateral involvement [7, 8]. The ILD-GAP index proposed by Ryerson et al. [9] was used to estimate mortality risk in patients with chronic ILD based on ILD clinical diagnosis, sex, age, %FVC, and %DLco, and was classified into stages I–III.

The study outcomes were the occurrence of an AE-criteria event within 1 month after influenza diagnosis, in-hospital mortality, and overall survival starting from the date of influenza diagnosis. In patients with AE-criteria events, we additionally recorded the time interval from influenza diagnosis to event onset, as well as the time interval from event onset to death.

### Statistical analysis

Survival analyses were conducted using the Kaplan–Meier method, with survival curves compared using the log-rank test. Hazard ratios (HR) and 95% confidence intervals (CIs) were estimated using the Cox proportional hazards model. Continuous and categorical variables were compared using the Mann–Whitney U test and the chi-square or Fisher’s exact tests, respectively. Statistical significance was defined as a two-sided *p* value < 0.05. Statistical analyses were conducted using EZR ver.4.0.0 (Saitama Medical Center, Jichi Medical University, Saitama, Japan), a graphical user interface for R (R Foundation for Statistical Computing, Vienna, Austria) [10].

## Results

### Patient population

A total of 927 patients with ILD were hospitalized at Toranomon Hospital during the study period. Of these, 47 (5.07%) were admitted with laboratory-confirmed influenza.

**Fig. 1:**
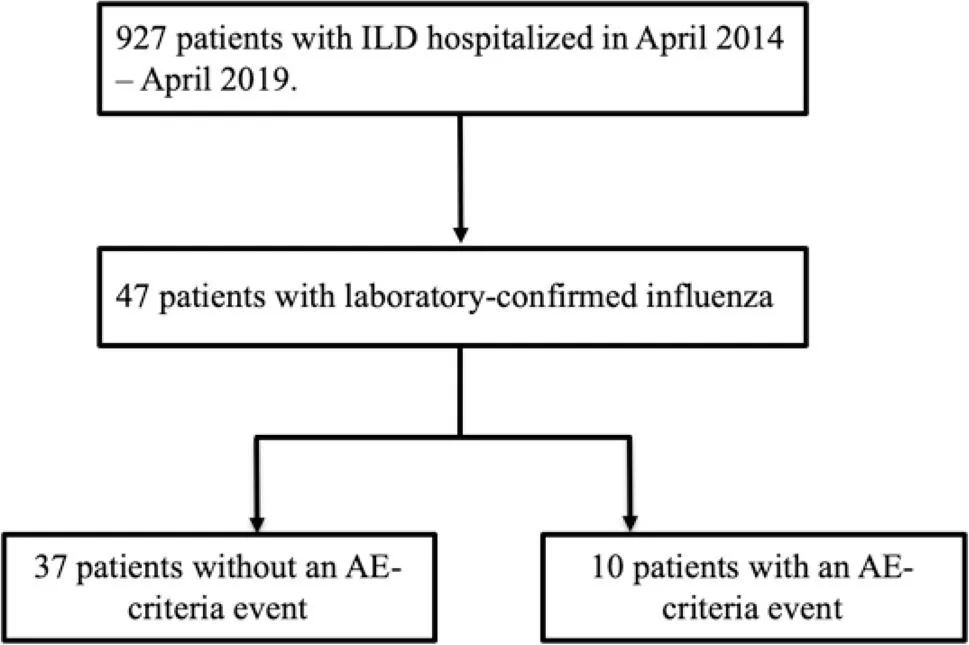
Flow diagram of patient selection. Abbreviations: AE, Acute exacerbation; ILD, interstitial lung disease

Influenza was confirmed by rapid influenza diagnostic tests in all 47 patients; however, influenza PCR testing was not performed, and testing for other respiratory viruses was not routinely conducted during the study period. Among the 47 patients, 10 (21.3%) developed AE-criteria events within 1 month after influenza diagnosis (Fig. 1).

### Baseline characteristics

Patient characteristics are presented in Table 1. The 47 patients hospitalized for influenza infection included 31 males (66.0%), and the median age was 76 years (interquartile range [IQR] 66.5–82.5). Of these 47 patients, 10 (21.3%) developed AE-criteria events. Compared with patients without AE-criteria events, those with AE-criteria events had higher serum levels of lactate dehydrogenase (LDH), KL-6, SP-D, and SP-A at influenza diagnosis. Baseline lung function was lower in the AE-criteria event group, including %FVC (70.0% vs. 88.0%), and ILD-GAP stage was also higher in this group.

**Table 1:** Baseline characteristics of patients with and without AE following influenza infection

Categories	All patients (*n* = 47)	No AE-criteria event (*n* = 37)	AE-criteria event (*n* = 10)	*P* Value^*^
Age^**^	76 (66.5–82.5)	76.0 (65.0–83.0)	74.5 (70.5–80.8)	0.805
Sex: Male / Female	31 / 16	25 / 12	6 / 4	0.716
BMI^**^	21.6 (18.7–24.5)	22.3 (18.7–24.5)	20.3 (18.7–24.3)	0.603
Smoking status:				
Never / Current or former	22/ 25	18/ 19	4/ 6	0.730
Brinkman index^**^	15.0 (0-770)	15.0 (0-800)	163.5 (0-575)	0.945
Medications for underlying ILD				
Anti-fibrotic agents	5	4	1	1.000
Steroids	26	20	6	1.000
Immunosuppressants	6	6	0	0.317
Past history of ILD-AE (+ / −)	17 / 30	14 / 23	3 / 7	0.727
Vaccination against influenza (+ / −)	13 / 34	12 / 25	1 / 9	0.244
Laboratory data				
BNP (pg/mL) ^**^	70.5 (32.0-129.0)	92.85 (31.0-217.1)	37.5 (32.5–65.2)	0.400
CRP (mg/dL) ^**^	2.6 (1.0-6.2)	2.50 (1.0-5.9)	4.55 (1.7–12.5)	0.140
LDH (IU/L) ^**^	254 (211.0-333.3)	223.0 (208.0-296.8)	379.0 (298.8-431.8)	0.001
KL-6 (U/mL) ^**^	752.0 (469.0-1331.0)	539.0 (429.0-778.5)	1337.0 (1030.5-1597.5)	0.003
SP-D (ng/mL) ^**^	138.2 (78.8–223.0)	102.0 (48.2-164.5)	269. 6(175.3-366.3)	0.001
SP-A (ng/mL) ^**^	59.2 (47.0-104.6)	51.3 (45.5–75.2)	113.9 (77.6-148.3)	0.032
Pulmonary function test				
VC (L) ^**^	2.50 (2.1–3.1)	2.66 (2.2–3.2)	2.11 (1.9–2.3)	0.048
VC, %predicted (%)^**^	82 (66.2–100.0)	86.5 (72.3-101.5)	67.0 (63.0–72.0)	0.073
FVC (L) ^**^	2.43 (1.9-3.0)	2.56 (1.9–3.1)	2.1 (1.4–2.3)	0.263
FVC, %predicted (%) ^**^	80.5 (66.9–98.8)	88.0 (70.5–104.0)	70.0 (60.0–72.0)	0.041
DLco (mL/min/mmHg) ^**^	9.06 (6.5–12.2)	9.94 (7.0-13.6)	8.47 (6.3–9.5)	0.723
DLco, %predicted (%)^**^	59 (41.0–70.0)	58.5 (42.5–70.3)	60.0 (35.0–70.0)	0.348
ILD-GAP index ^**^	3 (2.0–4.0)	3 (2.0–4.0)	4 (3.25-6.0)	0.106
ILD-GAP stage ^**^	1 (1.0–2.0)	1 (1.0–2.0)	2 (1.75-3.0)	0.010
Influenza type				
A / B / unknown^***^	28 / 17 / 2	23/ 13 / 1	5/ 4 / 1	0.574
HRCT pattern				0.545
UIP	20	14	6	
Probable UIP	4	3	1	
indeterminate for UIP	1	1	0	
alternative diagnosis	22	19	3	
Clinical diagnosis				1.000
IPF	11	8	3	
IIP other than IPF	14	11	3	
CTD-ILD	10	8	2	
drug-induced ILD	3	3	0	
fibrotic HP	3	2	1	
other	6	5	1	

*Abbreviations*: *AE* acute exacerbation, *ILD* interstitial lung disease, *BMI* body mass index, *BNP* B-type natriuretic peptide, *HP* hypersensitivity pneumonia, *CRP* C-reactive protein, *CTD* Connective Tissue Disease, *ILD* Interstitial Lung Disease, *LDH* lactate dehydrogenase, *KL-6* Krebs von den Lungen-6, *SP-A* surfactant protein A, *SP-D* surfactant protein D, *VC* vital capacity, *FVC* forced vital capacity, *DLco* diffusing capacity of the lung for carbon monoxide, *GAP* gender–age–physiology, *HRCT* high-resolution computed tomography, *UIP* usual interstitial pneumonia, *%predicted* percent predicted

* *P* value for the comparison between the non-AE group and the AE group

** median (interquartile range)

*** Two patients were diagnosed with influenza based on rapid diagnostic tests performed at other hospitals, but the type remains unknown

### Patients with AE

Among the 47 patients, 10 developed AE-criteria events. The median interval from influenza diagnosis to event onset was 5.0 days (IQR, 1.0–12.5 days). The in-hospital mortality rate in these patients was 30.0% (3/10 cases). In non-survivors, the median time from event onset to death was 22 days (range 4–36 days). Microbiological investigations were performed according to the judgment of the treating physicians. Sputum cultures were performed in 9 of the 10 patients, and microorganisms were isolated from 3 patients. The isolated microorganisms were *Chryseobacterium indologenes*, *Stenotrophomonas maltophilia*, and *methicillin-resistant Staphylococcus aureus*, one in each patient. Blood cultures were performed in all patients and were negative in all cases. Urinary antigen tests for *Streptococcus pneumoniae* and *Legionella pneumophila* were performed in three patients were all negative. BAL was not performed in any patient. Empirical antibiotics were administered to all patients with AE-criteria events.

**Fig. 2:**
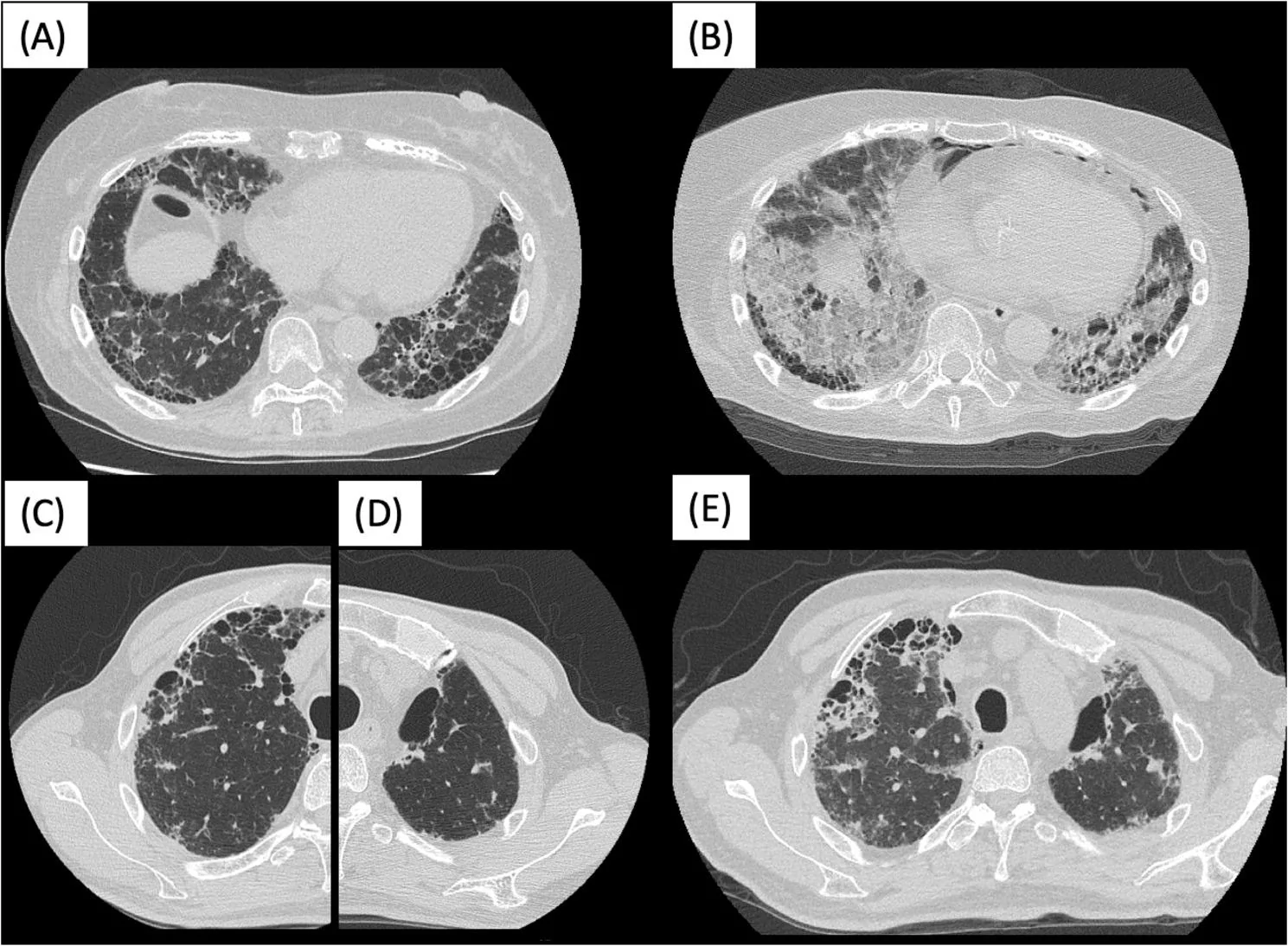
Representative HRCT images before and at the time of an AE-criteria event. Case 1 (diffuse pattern). **A** Baseline axial HRCT image from a 73-year-old woman with rheumatoid arthritis-associated ILD showing pre-existing fibrotic changes. **B** HRCT obtained at the onset of the AE-criteria event, 25 days after laboratory-confirmed influenza diagnosis, demonstrates newly developed bilateral ground-glass opacities and consolidation superimposed on the background fibrosis, consistent with a diffuse pattern. The patient died 4 days after the onset of the AE-criteria event. Case 2 (multifocal pattern). **C**, **D** Baseline axial HRCT images of the right and left lungs from a 72-year-old man with idiopathic pulmonary fibrosis showing pre-existing fibrotic changes. **E** HRCT obtained at the onset of the AE-criteria event, 3 days after laboratory-confirmed influenza diagnosis, demonstrates newly developed bilateral ground-glass opacities with a multifocal pattern. The patient improved following treatment. Abbreviations: AE, acute exacerbation; HRCT, high-resolution computed tomography; ILD, interstitial lung disease; IPF, idiopathic pulmonary fibrosis

At the time of the AE-criteria events, all 10 patients had new bilateral ground-glass opacities and/or consolidation superimposed on pre-existing ILD. The HRCT pattern was bilateral ground-glass opacities alone in 6 cases and mixed ground-glass opacities and consolidation in 4 cases; no cases had consolidation alone. The distribution of new opacities was multifocal in 5 cases and diffuse in 5 cases; no cases had a peripheral pattern. Representative HRCT images are shown in Fig. 2.

### Antiviral and steroid therapy

The antiviral drugs used in the enrolled patients are summarized in Table 2. Peramivir use occurred more frequently in the event group than in the non-event group (8/10 [80.0%] vs. 12/37 [32.4%], *p* = 0.011). There were no significant differences in oseltamivir, laninamivir, or baloxavir use. All patients with AE-criteria events received intravenous methylprednisolone pulse therapy (mPSL 1,000 mg/day for 3 days) followed by oral maintenance therapy with prednisolone 0.5–1.0 mg/kg/day, along with empirical antibiotic therapy. One patient received additional cyclosporine therapy.

**Table 2:** Antiviral drugs

Categories	No AE-criteria event , *n* = 37 (%)	AE-criteria event, *n* = 10 (%)	*P* Value
Antiviral drugs			
Peramivir	12 (32.4%)	8 (80.0%)	0.011
Oseltamivir	21 (56.8%)	2 (20.0%)	0.072
Baloxavir	1 (2.7%)	0 (0%)	1.000
Laninamivir	3 (8.1%)	0 (0%)	1.000

*Abbreviations*: *AE* acute exacerbation

### Seasonal influenza vaccination

Seasonal influenza vaccination was associated with a numerically lower incidence of AE-criteria events (7.7% [1/13] in vaccinated patients vs. 26.5% [9/34] in unvaccinated patients). However, this difference was not statistically significant.

### Survival outcomes

**Fig. 3:**
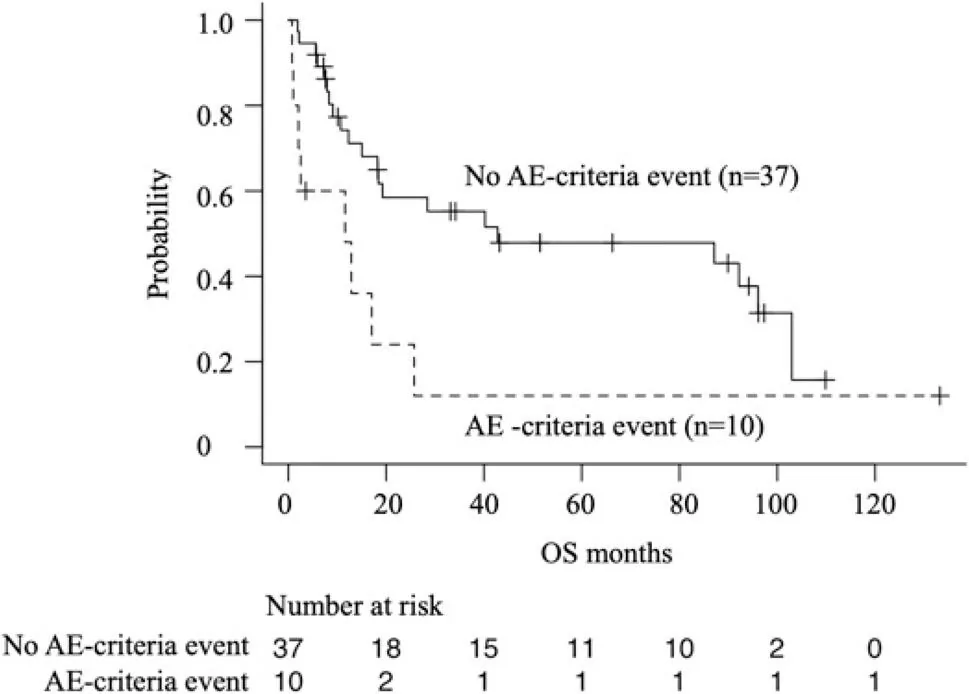
Overall survival after influenza diagnosis. The symbols on the scale indicate censored data. Patients alive at the time of the analysis were censored based on the last recorded date on which their survival was confirmed. Kaplan–Meier analysis suggested shorter overall survival in the AE-criteria event group than in the non-event group in unadjusted analysis (log-rank test, *p* = 0.032). Abbreviations: AE, acute exacerbation; OS: Overall survival

The median observation period of the entire cohort was 18.3 months. Kaplan–Meier analysis suggested shorter overall survival time from influenza diagnosis in the AE-criteria event group than in the non-event group in unadjusted analysis (log-rank test, *p* = 0.032) (Fig. 3). The median survival times were 11.6 months in the event group and 42.8 months in the non-event group. The respective 30, 60, and 90-day mortality rates were 20%, 20%, and 40% in the event group, compared with 0%, 2.8%, and 5.6% in the non- event group. In the univariate Cox proportional hazards model, AE-criteria events were associated with shorter overall survival (HR 2.42, 95% CI 1.05–5.54, *p* = 0.037).

## Discussion

The present study aimed to identify the incidence, clinical characteristics, and prognosis of AE-criteria events after laboratory-confirmed seasonal influenza in hospitalized patients with ILD at our center. AE-criteria events occurred in 21.3% of hospitalized ILD patients with influenza, developed a median of 5.0 days after influenza diagnosis, and were associated with high mortality. As previous studies directly addressing AE-criteria events after influenza in patients with ILD are limited, these findings provide important additional data regarding this serious clinical event.

The revised definition proposed by Collard et al. includes both idiopathic and triggered AE, including infection-triggered events [1]. However, this framework does not necessarily allow definite attribution of acute respiratory worsening to influenza-triggered AE. In patients with confirmed influenza, acute respiratory worsening may therefore represent influenza viral pneumonia, influenza-associated acute respiratory distress syndrome (ARDS), secondary bacterial pneumonia, triggered AE-ILD, or overlapping processes. This distinction is difficult because influenza pneumonia can also show bilateral ground-glass opacities, consolidation, or mixed opacities on CT and may progress to ARDS or diffuse alveolar damage [11–13].

In the present study, all AE-criteria events showed new bilateral ground-glass opacities and/or consolidation on pre-existing ILD, fulfilling the radiological criteria for AE-ILD. New opacities showed multifocal or diffuse distributions in all cases, without a peripheral pattern. Although deaths were numerically more frequent in the diffuse pattern group, consistent with previous AE-IPF studies [8], this finding should be interpreted cautiously because of the small sample size. HRCT findings alone could not determine whether the acute abnormalities represented influenza viral pneumonia, triggered AE-ILD, bacterial superinfection, or overlapping processes. Sputum cultures yielded microorganisms in three patients, including organisms that may cause pneumonia in immunocompromised hosts. Although these isolates may have represented colonization and no pathogen-directed antimicrobial therapy was administered beyond empirical antibiotics, bacterial superinfection could not be completely excluded in these patients. Thus, these cases should be interpreted as influenza-related acute respiratory worsening fulfilling AE criteria, rather than as definite influenza-triggered AE-ILD.

The AE-criteria events occurred early after influenza diagnosis, with a median interval of 5.0 days, and the in-hospital mortality rate was 30%. This mortality rate was within the previously reported range for AE-IPF or AE-ILD, although direct evidence regarding seasonal influenza-related AE-criteria events remains limited [1, 3, 14].

Patients with the AE-criteria event had more advanced disease at baseline. Compared with the non-event group, the event group had lower VC and %FVC and higher ILD-GAP stage. Poor lung function has been associated with an increased risk of AE-criteria event in fibrotic interstitial lung disease cohorts [15, 16]. Multivariate analysis was not conducted due to the small number of events. Therefore, these findings should be interpreted as differences between groups, rather than as independent risk factors for AE-criteria events. LDH, KL-6, SP-D, and SP-A levels were higher at the time of influenza diagnosis in the AE group. Given the short interval to event, these elevations may reflect early lung injury preceding the event [14, 16]. Although AE-criteria event was more common in patients with UIP/probable UIP patterns and in those with IPF or fibrotic HP, these subgroup findings should be interpreted descriptively because of the small number of events. In unadjusted Kaplan–Meier analysis, overall survival appeared shorter in the AE-criteria event group, suggesting that AE-criteria events after influenza may be associated with subsequent prognosis as well as short-term mortality. However, baseline disease severity may also have contributed to this difference. Because patients in the AE-criteria event group tended to have poorer lung function and more advanced ILD-GAP stages, their shorter survival may have been influenced by the severity of the underlying ILD as well as the AE-criteria event itself.

Systemic corticosteroid therapy for influenza is generally not recommended because current guidelines do not support its routine use, while observational studies have suggested an increased risk of mortality and secondary infections [17, 18]. High-dose systemic corticosteroids for AE of ILD are widely used in clinical practice. However, formal guidelines remain limited and are primarily based on AE of IPF. International guidelines currently provide only weak recommendations for corticosteroid therapy for AE of IPF [19–21]. A frequently used regimen involves the administration of intravenous methylprednisolone (500–1000 mg/day for 3 days), followed by oral prednisolone (0.5–1.0 mg/kg/day) using a tapered dosage schedule [20]. Empirical antibiotic therapy is also used because infection is a potential precipitating factor, and is often difficult to rule out at onset. At our hospital, all patients with AE-criteria events received antiviral therapy for influenza, empirical antibiotics, and high-dose corticosteroid pulse therapy, followed by maintenance prednisolone. Despite treatment, mortality remained high, and non-survivors exhibited a prolonged clinical course (median 22 days from AE onset to death) [1, 14]. Peramivir use occurred more frequently in the event group, but this likely reflects confounding by indication as intravenous peramivir is often selected for clinically severe patients or those unable to take oral or inhaled agents. Therefore, this finding should not be interpreted as an effect of peramivir itself.

Vaccinated patients had a numerically lower incidence of AE-criteria events, but the study was not of sufficient power to evaluate any preventive effects. Therefore, this finding should be interpreted as descriptive only. Considering the high mortality rate of AE-criteria events and the increased risk in patients with baseline pulmonary dysfunction, annual influenza vaccination and reinforced infection-prevention measures during respiratory viral seasons should be considered for high-risk patients, as recommended in the clinical practice guidance for IPF/progressive pulmonary fibrosis [4, 5].

### Research limitations

This study has several limitations. The sample size was small, and the study followed a single-center, retrospective design, which may have introduced bias. Furthermore, because of the small sample size and limited number of outcome events, multivariable analysis was not performed. Only 47 patients with ILD and laboratory-confirmed influenza were included, 10 patients developed AE-criteria events, and 3 patients died during hospitalization. Therefore, the findings should be considered exploratory and hypothesis-generating. Since this study included only hospitalized patients with a confirmed diagnosis of influenza, these findings cannot be generalized to all patients with concurrent ILD and influenza, including those with mild symptoms who were treated on an outpatient basis. In addition, vaccination status among the remaining hospitalized ILD patients without confirmed influenza was not systematically collected. Therefore, we could not compare vaccination rates between patients with and without influenza or evaluate vaccine effectiveness. Furthermore, because the sensitivity of rapid influenza diagnostic tests is limited, their use may have resulted in some cases being missed [22, 23]. PCR testing was not performed, and viral load, viral subtype, and viral co-infections were not assessed. Therefore, diagnostic misclassification could not be completely excluded. Lastly, it was not possible to completely rule out bacterial superinfection in all cases. Microbiological tests, BAL, and tests for other respiratory pathogens were not performed systematically, because this was a retrospective study based on routine clinical practice. In addition, severe influenza pneumonia and influenza-associated ARDS could not be clearly separated from AE-criteria events.

## Conclusion

In the present study, AE-criteria events occurred in approximately one fifth of hospitalized patients with ILD and laboratory-confirmed influenza. AE-criteria events commonly developed within a few days and were associated with high mortality. Although a tendency toward lower baseline lung function was observed in the AE-criteria event group, this small-scale retrospective cohort study was unable to determine whether this was independently associated with AE-criteria events. Larger multicenter studies are therefore required to confirm these exploratory findings, and further studies are needed to refine prevention and management strategies. These findings suggest that careful monitoring for AE-criteria events and early clinical evaluation may be beneficial in hospitalized patients with ILD following laboratory-confirmed influenza, particularly those with impaired baseline lung function.

## Data Availability

The datasets used and/or analyzed in this study are available upon reasonable request by contacting the corresponding author.

## Abbreviations

AE: Acute exacerbation
ARDS: Acute respiratory distress syndrome
BMI: Body mass index
CI: Confidence interval
DLco: Carbon monoxide diffusion capacity
FVC: Forced vital capacity
GAP: Gender–age–physiology
HRCT: High-resolution computed tomography
IIP: Idiopathic interstitial pneumonias
ILD: Interstitial lung disease
IPF: Idiopathic pulmonary fibrosis
IQR: Interquartile range
IRB: Institutional Review Board
KL-6: Krebs von den Lungen-6
LDH: Lactate dehydrogenase
Mpsl: Methylprednisolone
SP-A: Surfactant protein A
SP-D: Surfactant protein D
VC: Vital capacity
%FVC: Percent predicted forced vital capacity
%VC: Percent predicted vital capacity
